# Cannabinoid Receptor 2 Blockade Prevents Anti-Depressive-like Effect of Cannabidiol Acid Methyl Ester in Female WKY Rats

**DOI:** 10.3390/ijms24043828

**Published:** 2023-02-14

**Authors:** Danielle Hen-Shoval, Lital Moshe, Talia Indig-Naimer, Raphael Mechoulam, Gal Shoval, Gil Zalsman, Natalya M. Kogan, Aron Weller

**Affiliations:** 1 Psychology Department, Bar-Ilan University, Ramat Gan 5290002, Israel; 2 Gonda Brain Research Center, Bar-Ilan University, Ramat Gan 5290002, Israel; 3 Institute for Drug Research, Medical Faculty, Hebrew University, Jerusalem 9112002, Israel; 4 Geha Mental Health Center, Petah Tiqva 4910002, Israel; 5 Sackler Faculty of Medicine, Tel Aviv University, Tel Aviv 6997801, Israel; 6 Princeton Neuroscience Institute, Princeton University, Princeton, NJ 08540, USA; 7 Division of Molecular Imaging and Neuropathology, Department of Psychiatry, Columbia University, New York, NY 10032, USA; 8 Institute of Personalized and Translational Medicine, Molecular Biology, Ariel University, Ariel 4070000, Israel

**Keywords:** major depression disorder, endocannabinoid system, cannabidiolic acid methyl ester, genetic animal models of depression, Wistar–Kyoto (WKY), Forced swim test (FST)

## Abstract

The pathophysiology of major depressive disorder (MDD) is diverse and multi-factorial, yet treatment strategies remain limited. While women are twice as likely to develop the disorder as men, many animal model studies of antidepressant response rely solely on male subjects. The endocannabinoid system has been linked to depression in clinical and pre-clinical studies. Cannabidiolic Acid-Methyl Ester (CBDA-ME, EPM-301) demonstrated anti-depressive-like effects in male rats. Here, we explored acute effects of CBDA-ME and some possible mediating mechanisms, using a depressive-like genetic animal model, the Wistar–Kyoto (WKY) rat. In Experiment 1, Female WKY rats underwent the Forced swim test (FST) following acute CBDA-ME oral ingestion (1/5/10 mg/kg). In Experiment 2, Male and female WKY rats underwent the FST after injection of CB1 (AM-251) and CB2 (AM-630) receptor antagonists 30 min before acute CBDA-ME ingestion (1 mg/kg, males; 5 mg/kg, females). Serum levels of Brain-Derived Neurotrophic Factor (BDNF), numerous endocannabinoids and hippocampal Fatty Acid Amide Hydrolase (FAAH) levels were assessed. Results indicate that females required higher doses of CBDA-ME (5 and 10 mg/kg) to induce an anti-depressive-like effect in the FST. AM-630 blocked the antidepressant-like effect in females, but not in males. The effect of CBDA-ME in females was accompanied by elevated serum BDNF and some endocannabinoids and low hippocampal expression of FAAH. This study shows a sexually diverse behavioral anti-depressive response to CBDA-ME and possible underlying mechanisms in females, supporting its potential use for treating MDD and related disorders.

## 1. Introduction

Major depressive disorder (MDD) is a leading cause of disability worldwide [1]. The proportion of people living with depression is estimated to be 4.4% (322 million people) of the world’s population [2]. Although depression can affect anyone at any point in their lives, it is more common in women than men [3]. In spite of this, female depression and sex differences in depression have received considerably less attention in psychopharmacological research [4]. While there are many effective antidepressants available for treating depression [5], an estimated 40% of patients remain symptomatic [6], and a large portion of those who are responsive suffer from a range of possible side effects, such as dry mouth, gastrointestinal dysregulations, hepatotoxicity, sexual dysfunction, etc. [7]. Hence, innovation in psychopharmacology is crucially needed [5].

One possible molecular target for innovative psychopharmacology treatment is the endocannabinoid system (ECS). Widely distributed throughout the brain, the ECS is involved in the neurobiology of depression [8,9], and its activity may be modified by exogenous cannabinoids [10].

While there is emerging evidence for the effectiveness of Cannabidiol (CBD), the main non-psychomimetic component of *Cannabis sativa* L., in depression [11,12], other cannabinoids are still being explored.

In particular, Cannabidiolic acid (CBDA), the precursor of CBD, has been evaluated at doses 1000 times lower than the effective doses of CBD [13,14]. However, CBDA is known to be unstable, undergoing rapid decarboxylation into CBD.

**Figure 1:**
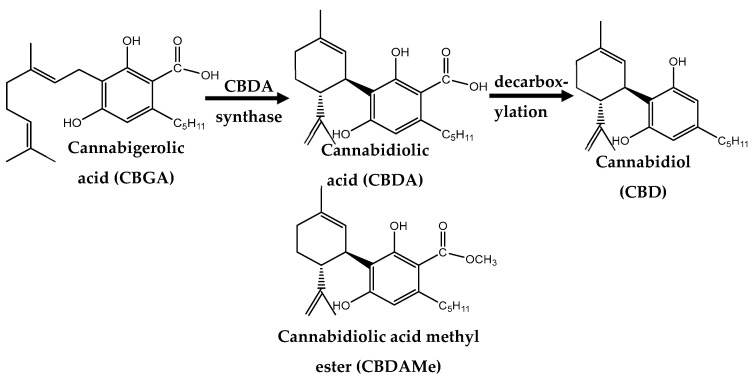
Conversion of cannabigerolic acid (CBGA), to cannabidiolic acid (CBDA), to cannabidiol (CBD) and CBDA stable analogue CBDA methyl ester (CBDA-ME) (ChemDraw Professional drawing).

More recently, the semi-synthetic analogue Cannabidiolic Acid Methyl-Ester (CBDA-ME; HU-580; EPM301) which is significantly more stable than CBDA was evaluated (Figure 1) [15].

CBDA-ME may potentially exert even more-potent effects. Recent studies in male rodents show that low doses of CBDA-ME are anxiolytic [15], anti-hyperalgesic [16] and anti-depressive [17]. Yet, its mechanisms of action are not clear, though they may resemble those of CBD.

**Figure 2:**
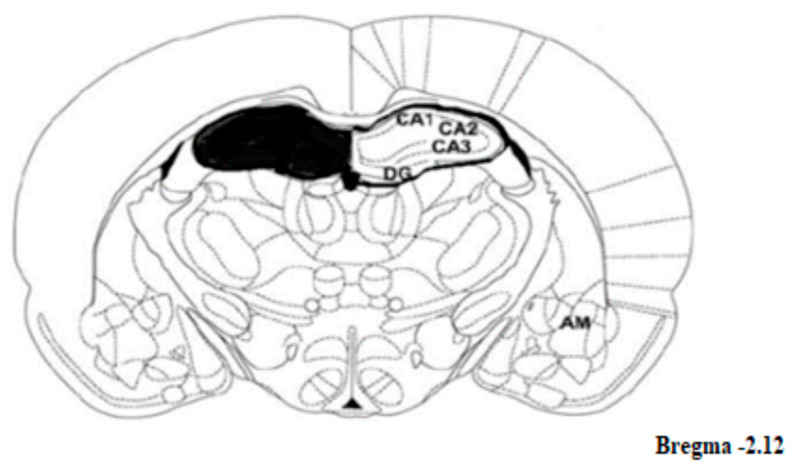
Schematic drawing of the rat hippocampus area that was taken—CA1, CA3 and the dentate gyrus (DG). Figure was taken from: http://labs.gaidi.ca/rat-brain-atlas/?ml=-2.5&ap=-3.8&dv=1.6, accessed on 15 July 2022.

Among the possible biological mechanisms underlying the effects of CBD are the involvement of Cannabinoid receptor 1 (CB1) [18], Cannabinoid receptor 2 (CB2) [19], immune system’s cytokines such as IL-4, IL-6, IL-10,TNF-α [20,21], Fatty Acid Amino Hydrolase (FAAH) [22] and brain derived neurotrophic factor (BDNF) levels [23]. As the hippocampal regions have been identified in the pathology of MDD [24,25], they are presumed to be involved in the anti-depressive-like effect of CBD (the sections taken by us are depicted in Figure 2) [26,27]. Wistar–Kyoto rats (WKY) are a valuable model for studying depression for their many depressive-like characteristics such as high activity level of the hypothalamic-pituitary-adrenal (HPA) axis in response to stress [28], increased behavioral immobility in the Forced Swim Test (FST) and comorbid anxiety-like behavior [29,30].

In the present study, we investigated whether acutely administered CBDA-ME has an anti-depressive-like effect in female WKY rats and whether or not it impacts the levels of corticosterone, pro-inflammatory cytokines, BDNF and FAAH, in the blood and brain.

**Figure 3:**
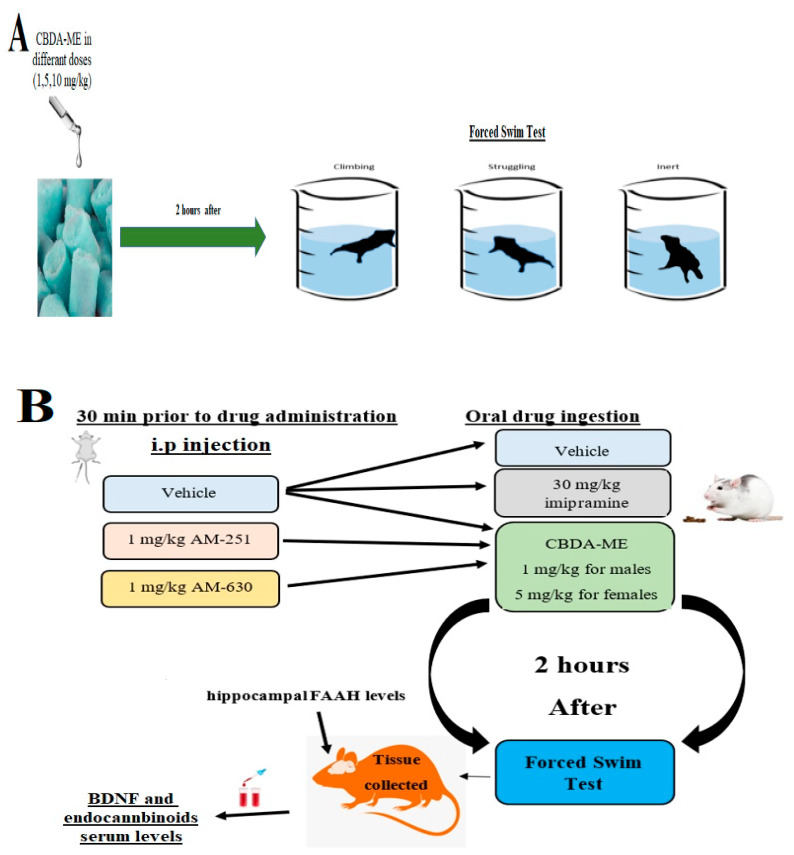
(**A**) Graphic description of the experimental procedure—Experiment 1. (**B**) Graphic description of the experimental procedure—Experiment 2.

To further understand the biological mechanisms of this compound, we also investigated whether the administration of CB1 and CB2 receptor antagonists inhibits CBDA-ME’s effects (for the experiment design see Figure 3). 

## 2. Results

### 2.1. Experiment 1

(a) FST in female WKY rats

**Figure 4:**
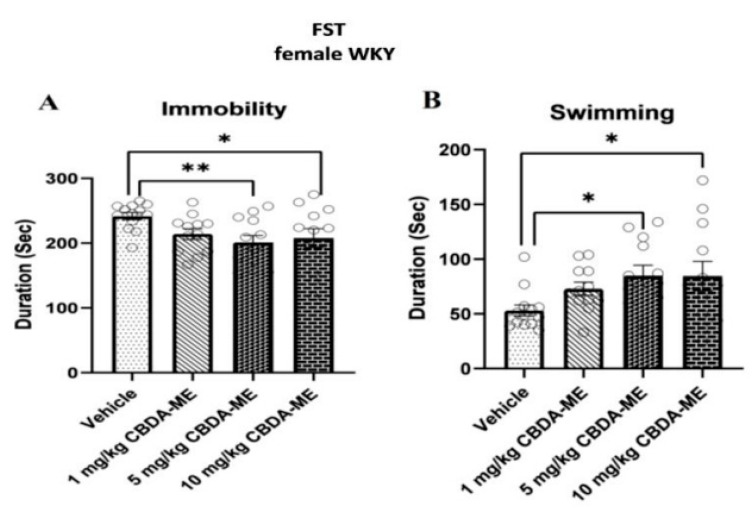
(**A**) Duration of immobility (mean + SEM) of female WKY rats (**B**) Duration of swimming (mean + SEM) of female WKY rats. Rats ingested either vehicle (n = 13) or 1 mg/kg CBDA-ME (n = 12), 5 mg/kg CBDA-ME (n = 12), 10 mg/kg CBDA-ME (n = 12). ** *p* < 0.01, * *p* < 0.05.

Swimming time was significantly prolonged, while the immobility time was significantly reduced in CBDA-ME-treated groups, at the concentrations 5 and 10 mg/kg, while 1 mg/kg was ineffective. A total of 5 mg/kg was an optimal, best-acting concentration (Figure 4).

(b) Corticosterone levels in blood

There was no significant difference between WKY treated with different doses of CBDA-ME- and vehicle-treated WKYs. Naïve WKY rats had significantly lower corticosterone levels in their blood than rats that underwent FST (see Figure S1 in the Supplementary).

(c) Cytokine levels in blood

IL-1b, IL-2, IL-4, IL-6, IL-10, TNF-a, and IFN-gamma levels did not differ between naïve, vehicle-injected and 1, 5 and 10 mg/kg CBDA-ME-treated WKY rats (Table S1 in Supplementary).

### 2.2. Experiment 2

(a) FST in male WKY rats

The 1 mg/kg of CBDA-ME-treated WKY males floated significantly less and swam significantly more than vehicle-treated WKYs (Supplementary Table S2). These findings confirm and replicate our previous findings in male WKY rats [17].

The CB1 and CB2 antagonists did not significantly affect the impact of CBDA-ME on swimming and immobility times.

(b) FST-female WKY rats

**Figure 5:**
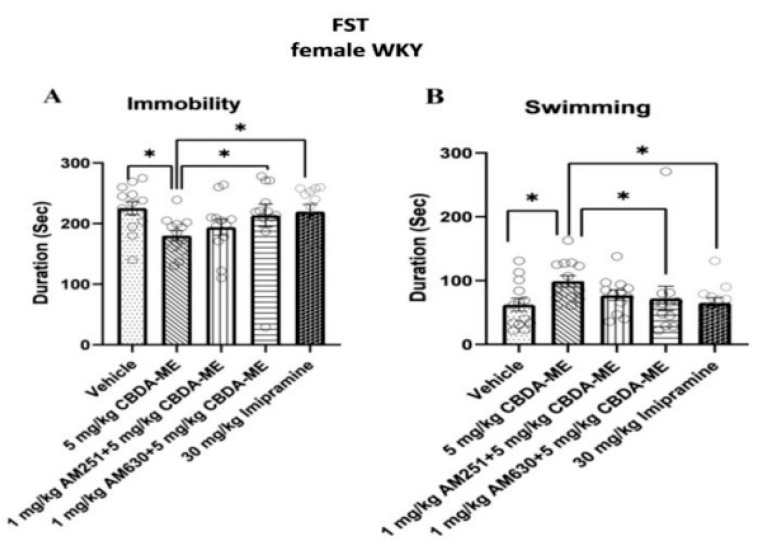
(**A**) Duration of immobility of female WKY rats (**B**) duration of swimming (mean + SEM) of female WKY rats. Rats received either vehicle (n = 13) or 5 mg/kg CBDA-ME (n = 13), 1 mg/kg AM251 + 5 mg/kg CBDA-ME (n = 12), 1 mg/kg AM630 + 5 mg/kg CBDA-ME (n = 12), 30 mg/kg Imipramine (n = 12). * *p* < 0.05.

The 5 mg/kg CBDA-ME-treated WKY female rats floated significantly less and swam significantly more than vehicle-treated animals. AM630 treatment interfered with the effect of CBDA-ME, suggesting some involvement of the CB2 receptor (Figure 5).

(c) Sex differences

MANOVA did not produce a significant sex X drug interaction, although overall females swam less (*p* < 0.05) and tended to be more immobile (*p* < 0.085) than males (data shown in Figure 5 [females] and Table S2 in Supplementary).

(d) BDNF measurement in blood

**Figure 6:**
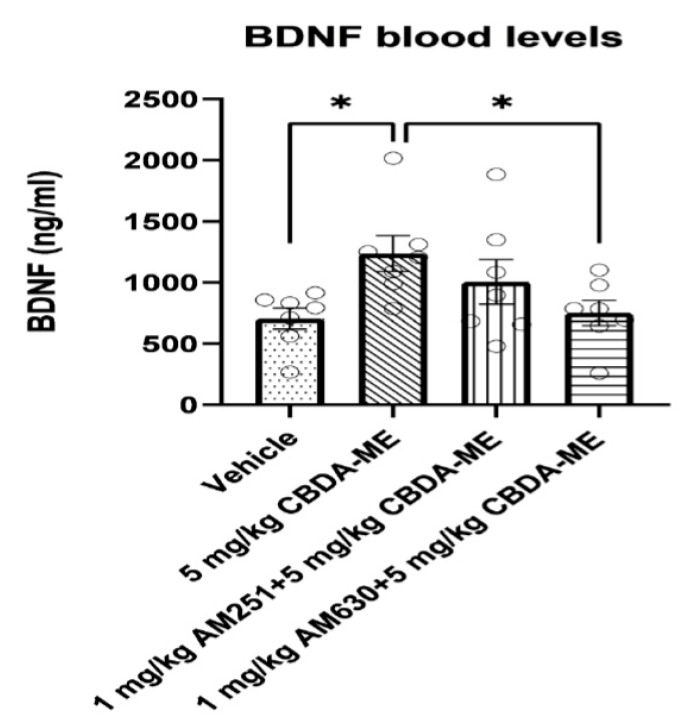
BDNF blood level (mean + SEM) of female WKY rats. Rats received either vehicle (n = 7) or 5 mg/kg CBDA-ME (n = 7), 1 mg/kg AM251 + 5 mg/kg CBDA-ME (n = 7), 1 mg/kg AM630 + 5 mg/kg CBDA-ME (n = 7). * *p* < 0.05.

The 5 mg/kg CBDA-ME-treated WKY female rats had significantly higher levels of serum BDNF compared with vehicle-treated animals. AM630 treatment interfered with the effect of CBDA-ME (Figure 6).

(e) Endocannabinoid levels in blood

**Figure 7:**
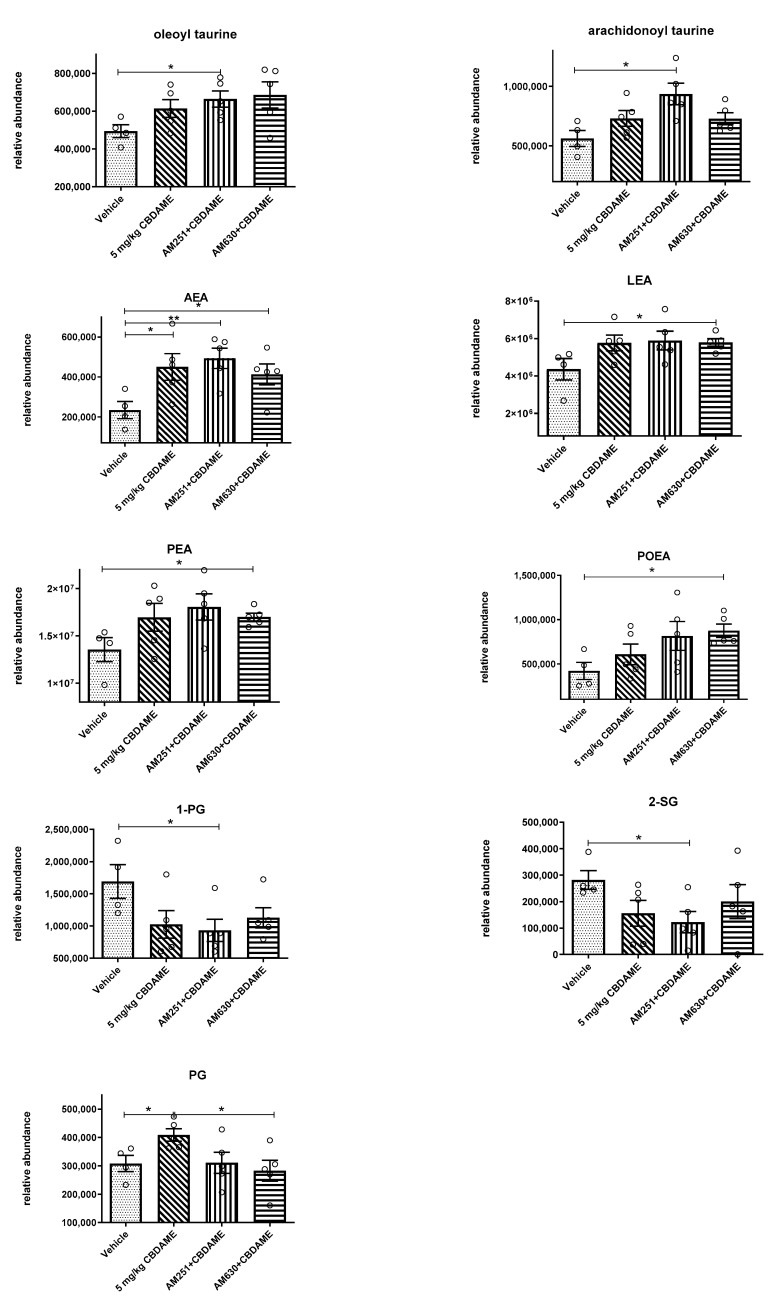
Selected endocannabinoids blood level (mean + SEM) of female WKY rats. Rats received either vehicle (n = 4) or 5 mg/kg CBDA-ME (n = 5), 1 mg/kg AM251 + 5 mg/kg CBDA-ME (n = 5), 1 mg/kg AM630 + 5 mg/kg CBDA-ME (n = 5). * *p* < 0.05; ** *p*< 0.01.

Of the 31 endocannabinoid-like molecules (LauroylEA, MEA, PEA, 2-PG, 1-PG, LEA, OEA, OG, EPEA, O-Ala, 2-LG, 1-LG, OS, DHEA, DEA, DTEA, DEEA, DSEA, OTau, Oleamide, HEA, AEA, 2-AG, 1-AG, ARA-Tau, 1-POG, 2-POG, 2-SG, 1-SG, POEA, PG), which were compared in control vs CBDA-ME-treated female rats and antagonists-treated group, we found some differences in endocannabinoid levels (Figure 7). When analyzed by an independent samples t-test, the ethanolamines (PEA, LEA, AEA, POEA) and also acyl-taurines (ARA-Tau and O-Tau) showed the same pattern: their levels in serum were higher in the CBDA-ME-treated group (though, for most of the compounds, insignificantly), and even higher in the CBDA-ME + AM-251-treated group and/or the CBDA-ME + AM-630 group (usually significantly). Both antagonists did not interfere with the effect of CBDA-ME on endocannabinoid levels elevation. The even better effect in the presence of antagonists is most probably part of some compensation mechanism of the body (as the receptors are blocked, the body tries to compensate for it by producing more of the ligand). The only two endocannabinoids in which the treatment by CBDA-ME significantly elevated their levels are AEA and PG. Of them, only the PG elevation can be significantly prevented by AM-630. The levels of 2-AG did not differ between the CBDA-ME-treated and vehicle-treated rats. The levels of 1-SG and 2-PG were significantly reduced by CBDA-ME treatment, with no influence on the antagonists in this effect.

All the rest of the endocannabinoids were not significantly changed after CBDA-ME treatment.

(f) FAAH expression in the hippocampus

**Figure 8:**
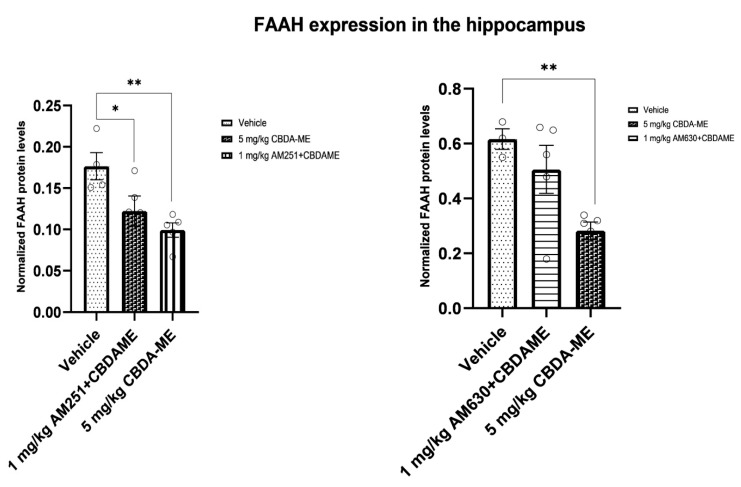
FAAH expression in the hippocampus (mean + SEM) of female WKY rats. Rats received either vehicle (n = 4) or 5 mg/kg CBDA-ME (n = 5), 1 mg/kg AM251 + 5 mg/kg CBDA-ME (n = 5), 1 mg/kg AM630 + 5 mg/kg CBDA-ME (n = 5). * *p* < 0.05, ** *p*< 0.01.

The 5 mg/kg CBDA-ME-treated WKY female rats had significantly lower hippocampal FAAH expression compared with vehicle-treated rats (Figure 8). This effect was significantly prevented by AM-630, but not by AM-251 (Figure 8, see Figure S2 in Supplementary for images of the western blot).

## 3. Discussion

One of the most striking statistics of MDD is that women are twice as likely to develop the disorder compared with men [30]. This sex difference in depression rates may exist, in part, due to particular biological and environmental risk factors experienced by men and women [31]. Research suggests that gender differences in biological processes and pharmacokinetics may affect response to antidepressants [32]. Despite these findings, many of the animal models used to study biological mechanisms of depressive symptoms or antidepressant response rely solely on male subjects, ignoring a critically important study population [3]. Accordingly, despite the sexual dimorphism present in the pathophysiology and etiology of MDD, sex differences in the WKY model are rarely investigated [33]. This highlights a great problem as knowledge regarding sex differences in depression is crucial to understanding differential antidepressant efficacy [3].

In the present study, we explored behavioral effects and possible underlying mechanisms of action of the synthetic analogue of cannabidiolic acid: CBDA-ME. In fact, we extended the work previously reported [16], by confirming the antidepressant effect of orally administrated CBDA-ME in females of a genetic animal model of depression; WKY rats. Furthermore, we proposed a possible cannabinoid-receptor-mediated biological mechanism for (at least part of) the acute antidepressant-like effect in females.

Although both 5 mg/kg and 10 mg/kg of CBDA-ME showed similar effects in the FST, the middle dose—5 mg/kg—was more potent in decreasing the duration of immobility in the FST. This finding establishes and corroborates the properties of CBDA-ME [14,15,34,35], specifically of its anti-depressive-like effect, as was previously found by Hen-Shoval et al. [16]. This anti-depressive-like effect is not only revealed in both sexes, but it appeared in response to a lower dose than the anti-depressive-like effect of CBD [36,37,38]. Since most of the study of antidepressant efficacy relies on males, this finding strengthens the validity of this compound as a novel antidepressant. The discovery of the potent dose in males and females, the path for examining the chronic effect of this compound is now open.

It should be noted that these results demonstrated a sexually diverse response to the drug treatment; the potent dose for males was five times lower than the potent dose for females [16]. This finding is in accordance with evidence in the literature suggesting that gender differences in biological processes and pharmacokinetics may affect response to antidepressants [32]. Moreover, this phenomenon has been demonstrated in other animal studies, showing that males and females are responsive to different doses of the same drug [39,40,41].

Cytokine levels are known to be altered in depression [42,43,44], thus we expected them to differ between the groups; however, in our study, no differences in cytokine levels were seen. This contradiction is possibly due to the fact that most studies examine cytokine response after injection of lipopolysaccharide (LPS) as an immune challenge [45,46]. In the present experiment, we did not use LPS injection as it was found to induce depressive-like behavior, expressed by increasing immobility time in the FST in mice [47]. Thus, it is possible that the reason that cytokine blood levels were not altered by CBDA-ME was the absence of an immune challenge, and not necessarily the CBDA-ME’s inability to change cytokine levels, as it is known that cannabis compounds such as CBD induce immune modulation [19,20].

In addition, we explored the possibility that the anti-depressive-like effect of CBDA-ME is, in part, due to reduced CORT blood levels in WKYs, that are known to have increased stress-induced CORT levels relative to other rat strains [48,49]. However, the CORT blood levels of the animals were similar in each group in our study; the CBDA-ME (1, 5, 10 mg/kg) and the vehicle. The only difference that was found was the higher CORT blood levels in the groups that underwent the FST compared with naïve rats, representing the dramatic effect of FST, inducing higher levels of blood CORT in the WKY [50,51,52]. While many studies have indicated that the ECS regulates the HPA axis activity under basal or stress-related conditions [53,54], there are some pre-clinical studies that reported that treatment with CBD increased CORT levels [11]. Future study is needed to explore this matter, with several time points post-FST [50].

Novel findings from the present study indicate two major observations. First, CBDA-ME induces acute anti-depressive-like effect where a common antidepressant such as imipramine does not. As evidence in the field demonstrates that chronic, but not acute treatment with the TCA imipramine (10–15 mg/kg/day for 13–28 days), reduces immobility in the FST in male WKY rats [55,56] and that WKY rats display resistance to sub-acute fluoxetine treatment (10 mg/kg 23.5, 5 and 1 h before the FST) [57], they are not only a good animal model of depressive-like behavior, but also may be useful as a model of treatment-resistant depression [33]. A general consensus is that a minimum of 3–6 weeks of continuous therapy at optimal dose is necessary to determine whether a patient is refractory to a particular antidepressant agent [58,59]. Therefore, the acute anti-depressive-like effect of CBDA-ME in this model is of even more extraordinary importance.

Secondly, there are sex differences in the mediation of CB2 receptors in the anti-depressive-like effect of CBDA-ME. Pretreatment with AM-630 blocked the anti-depressive-like effect in female, but not in male WKY rats. Moreover, the behavioral antidepressant-like effects of CBDA-ME were neither prevented nor mimicked by AM251 in males (1 mg/kg) or females (5 mg/kg), suggesting that the modulation of the CB1 receptors does not contribute to the acute antidepressant-like effects of CBDA-ME. These results are in line with a previous report of pretreatment AM-251 that did not modify the anti-depressive-like effect of CBD [37]. These results establish and emphasize the dimorphism of CBDA-ME in its anti-depressive-like effect.

Since male WKY rats were not responsive to the CB1 and CB2 antagonist, biological measurements were conducted in females only. In addition, because 30 mg/kg of Imipramine did not induce anti-depressive-like effect, this group was excluded from the analysis as well.

In an overview of the biological findings, several novel results need to be addressed. First, in line with the anti-depressive-like effect, we found that acute CBDA-ME treatment elevates BDNF blood levels. BDNF triggers multiple intracellular signaling cascades, which increase synaptogenesis and appear to play an important role in the behavioral effects induced by fast-acting antidepressants [23]. Although CBD, as other antidepressant medications [60,61], is known to increase BDNF levels [22], this effect was now shown, for the first time, using CBDA-ME treatment. Moreover, this effect was abolished in AM-630 pretreated groups.

Similarly, this was reflected in the serum endocannabinoids and hippocampal FAAH measurements. These measurements have shown additional abilities of CBDA-ME to alter endocannabinoid signaling by inhibiting FAAH activity. This was demonstrated by higher levels of FAAH in the hippocampus and lower AEA and other endocannabinoids’ blood levels in the vehicle compared to CBDA-ME.

As was reported before, lower levels of AEA and higher levels of FAAH are found in the frontal cortex and hippocampus of WKY compared to Wistar male rats without any behavioral manipulation [62]. Thus, it is possible that CBDA-ME treatment affected both of these elements, as established CBD treatment [63,64,65,66].

The role of endocannabinoids in depression is still not clear and is currently under investigation; different publications show antidepressant effects of different endocannabinoids. For example, PEA exerts pleiotropic actions including improving neuropathic pain, inhibiting food consumption, suppressing cancerogenic cell proliferation, being an antiepileptic and has behavioral effects by acting as an anxiolytic and antidepressant [67,68]. PEA levels, but also those of the congener oleoyl ethanolamide (OEA), were decreased in the hair of individuals who suffer from PTSD. The decrease of these endocannabinoid-like compounds was inversely correlated with the severity of PTSD symptoms in this population [69].

In addition, in the model of high-fat diet-induced depression, PEA, LEA and OEA were found to be down-regulated; this down-regulation is prevented by simvastatin, a lipid-lowering agent, and parallel its antidepressant effect [70].

In women with PTSD, greater circulating concentrations of AEA within the PTSD group were associated with lower depressive mood, confusion, and total mood disturbance [71]. In another study, reduced AEA and cortisol levels were found in the group with a positive MDD screening compared to individuals with low depressive symptomatology [72]. However, another study reported that traumatic stress during childhood and later in life as well as more severe depressive and physical stress symptoms were associated with elevated 2-AG, SEA, OEA, and PEA concentrations in human hair [73]. In our experiment, PG (palmitoyl glycine) levels were elevated in CBDA-ME-treated rats’ serum, an effect which was prevented by the CB2 antagonist AM-630. The effect of AM-251, a CB1 antagonist, was almost the same, although not statistically significant. The prevention of PG elevation is in line with the down-regulation of FAAH by CBDA-ME, as PG was previously found to be up-regulated in FAAH knockout mice, suggesting FAAH involvement in its metabolism. It is yet unclear why both antagonists can prevent the elevation in PG, although only AM-630 prevents the down-regulation of FAAH. Possibly additional, CB1-mediated mechanisms of PG biosynthesis exist, in addition to the prevention of its degradation by FAAH, which is probably mediated by CB2. As for the possible mechanism of action of this endocannabinoid in depression, PG was found to possess an anti-immobility action in the forced swimming test in mice [74], and it might also involve Ca influx regulation. PG was found to induce transient calcium influx in native adult dorsal root ganglion (DRG) cells and a DRG-like cell line (F-11) [75].

The role of Ca influx in depression is quite controversial. From one side, in some experiments where Ca reduction was observed in depressed conditions [76], Ca was found to be involved in the antidepressant effect of lateral habenula stimulation [77] and long-term imipramine treatment (48 h) increased Ca2 þ influx via NMDA receptors [78]. From the other side, some Ca channels are to be involved in depressive behavior, and Ca-channel blockades were found to have some antidepressant effects [79].

The ethanolamines and some acyl-amino acids serum level data are in-line with the compensation mechanisms. In AM-251 and AM-630-treated groups, anandamide, and some other endocannabinoids that work (at least partially) through CB1 and CB2 go up to compensate for the CB1 or CB2 inhibition. Thus, the role of AM-630 in endocannabinoid serum levels in this case may be dual: from one side it interferes with the possible partial activation of CB2 by CBDA-ME (and thus is supposed to prevent the elevation of endocannabinoids’ levels through this mechanism); however, from the other side, as it blocks the receptor, it can cause the elevation of endocannabinoids’ levels through the compensation mechanisms. The difference in the response of different ethanolamines levels to CBDA-ME treatment is probably a result of the difference of their affinity to CB1/CB2 and to FAAH.

In contrast to ethanolamines, no significant change was found in 2-AG serum levels, and in the levels of most of other acyl-glycerols. This can be explained by the investigation of the relative activity of monoacylglycerol lipase (MAGL) and FAAH enzymes, the major factors determining the functional role of 2-AG and AEA as endogenous analgesics. Della Pietra et al. (2022) [80] suggested that their activity is not equally present in the PNS and CNS. Indeed, endocannabinoid hydrolysis, MAGL and FAAH, are differentially active in the trigeminal ganglion, which is a part of the peripheral nociceptive system and in the brain areas [81]. Likewise, the levels of endocannabinoids in the periphery are expected to be non-equally present in favor of accumulated AEA, while the amount of 2-AG should be ultimately low due to the active degradation by MAGL [81]. Notably, this imbalance could be changed by the inhibition of MAGL activity, which sets the MAGL/2-AG axis as a highly tunable target for pharmacological interventions [80]. Perhaps measuring 2-AG in discrete brain areas would have revealed results in the current studies on animals, but we only measured blood 2-AG, a limitation of the present study.

We are aware that translation from our animal model’s results to human depression (and different types of depression) has to be examined carefully. Although FAAH is known to be involved in depression mechanisms in animal studies (the most recent examples [82,83]), much more investigation is needed to ensure the same mechanisms are relevant to humans. However, only a few clinical trials performed till now support the assumption that FAAH plays an important role in depression mechanisms. Research performed on the human genetic variant C385A (rs324420) of FAAH suggests that it enhances fear extinction learning [84]. A recent clinical trial on PTSD depressive patients has shown that the FAAH A385 carriers improved more under D-cycloserine treatment compared to non-carriers, particularly among participants who had MDD [85]. In another clinical trial, performed on people with SAD (social anxiety disorder), an inhibitor of FAAH, JNJ-42165279 significantly improved the Liebowitz Social Anxiety Scale (LSAS) total score from baseline to end of study. Treatment with JNJ-42165279 was associated with increases in plasma AEA, PEA, and OEA [86].

The dose of 5 mg/kg CBDA-ME, used by us, is completely safe. In other studies, much higher doses have been used chronically, without any sign of toxicity (see for example [87], where the doses of 20 mg/kg/day and 40 mg/kg/day have been used for 4 weeks without any toxic effect).

It should also be noted that CBDA-ME in our experiments was administered per os. Most of cannabinoids have extremely low bioavailability, and hardly 6–7% of the orally administered dose reach the bloodstream. The pharmacokinetics of CBDA-ME have not been evaluated yet, but the fact that oral doses have significant effect is optimistic.

Taking all this into account, CBDA-ME seems a very promising antidepressant treatment, which has no side-effects, acts acutely, and maybe can bridge the gap between the start of treatment and the start of action of regular antidepressants.

## 4. Materials and Methods

### 4.1. Animals

The study included WKY young-adult male and female rats, 70-days-old (mean weight: 200 and 350 g, respectively). The rats were provided by Bar-Ilan University’s colony. Rats were housed two per cage (38 × 21 × 18 cm.), in a temperature-controlled facility (22 + 1 °C), under a 12 h–12 h light: dark cycle (lights on at 07:00). Food and water were available ad libitum. The study protocol adheres to the guidelines of the Society for Neurosciences and was approved by the Institutional Animal Care and Use Committee.

### 4.2. Forced Swimming Test (FST)

The swimming test that was performed is similar to the one often described before [88] with minor modifications. We used a Plexiglas cylinder 45.5 cm tall, 25 cm diameter filled up to 30 cm with 24 ± 0.5 °C water. The animals were immersed in the cylinder for 5 min. Duration of immobility, swimming and struggling behavior were measured online using a stopwatch. At the end of each 5 s period during the test session, the scorer rated the rat’s behavior at that time, as one of the following three categories: (1) immobility—floating in the water without struggling, and making only those movements necessary to keep its head above water. (2) swimming—making active swimming motions, more than necessary to merely maintain the head above water, i.e., moving around the cylinder. (3) climbing—making active movements with forepaws in and out of the water, usually directed against the walls. FST measurement was double blind, conducted by two researchers. Behavior scoring was performed by a single researcher, who was blind to treatment conditions. After the test, rats were dried off, the cylinder cleaned, and water changed.

### 4.3. Drugs

Experiments 1 and 2: CBDA-ME was synthesized as previously described [89] (Figure 1) and was provided by Raphael Mechoulam’s laboratory. In order to prevent neophobia, the rats received a high-fat diet pellet with a 100-μL drop of ethanol 2 times during the week prior to the experiment (individually in a holding cage). For oral administration, CBDA-ME and Imipramine (Sigma–Aldrich, St. Louis, MO, USA) were dissolved in glass graduated tubes with different amounts of ethanol (in accordance with the various doses administrated to the animals). Vehicle, CBDA-ME and Imipramine solutions were prepared immediately before use.

Oral ingestion protocol: On the test day, each animal was placed in an individual cage and was given a pellet of high fat rodent diet (D12492 Research Diets, Inc. Rodent diet with 60% Fat, NJ, USA) laced with vehicle, CBDA-ME or Imipramine. The rats completed eating the pellet within 5 min without any need for coercion. Experiment 2: A selective CB1 receptor antagonist AM251 (1-(2,4-dichlorophenyl)-5-(4-methoxyphenyl)- 4-methyl-N-(1-piperidinyl)-1H pyrazole-3 carboxamide; Tocris Bioscience, UK) and a selective CB2 receptor antagonist AM630 (6-Iodo-2-methyl-1-[2-(4-morpholinyl)ethyl]-1H-indol-3-yl](4methoxyphenyl) methanone; Tocris, Bristol, UK) were initially dissolved in dimethyl sulphoxide (DMSO; SIGMA, St. Louis, MO, USA) and then diluted with 0.9% saline using TWEEN 80 (SIGMA, St. Louis, MO, USA). The final concentrations of DMSO: TWEEN 80: saline was 1:1:18. The drugs were administered IP.

### 4.4. Cytokines Assessment

Blood samples (collected 15 min after the end of the FST, 2 h after CBDA-ME administration) were centrifuged at 3000 rpm for 15 min, and then the supernatants were collected and set aside at −80 °C for serum cytokines analysis. Plasma inflammatory cytokines, including IFN-γ, IL-1β, IL-2, IL-6, tumor necrosis factor-alpha (TNF-α) and anti-inflammatory cytokines including IL-4, IL-10, were measured using a Luminex screen assay kit (R&D Systems, Minneapolis, MN, USA) and a Luminex analyzer (Luminex, Austin, TX, USA).

### 4.5. Corticosterone Measurement

Blood samples (collected 15 min after the end of the FST [50], 2 h after CBDA-ME administration) were centrifuged at 3000 rpm for 15 min, and the supernatants were collected and set aside at −80 °C for serum cytokines analysis. The samples were analyzed by CORT ImmunoAssay Enzo ADI-900-097 following the manufacturer’s instructions (Enzo Life Sciences, Plymoth meeting, PA, USA).

### 4.6. Brain Regions

In Experiment 2, rats were decapitated immediately after the FST and their brains were preserved at −80 °C. The hippocampal dentate gyrus (1.0 mm), CA1 and CA3 cortices (1.5 mm) samples were punched from the frozen brain slices (-20) in accordance with the rat brain atlas [90], using cylindrical brain punchers (Fine Science Tools; internal diameter, 1.0/1.5 mm). Tissues were immediately frozen on dry ice and stored (−80 °C) for later analysis (Figure 2).

### 4.7. BDNF Levels in Blood

The total amount of proteins in blood serum was determined for each animal’s supernatant, and BDNF levels were measured by a BDNF ELISA kit (RAB1138-1KT, Sigma, St. Louis, MO, USA) [91] following the manufacturer’s instructions.

Briefly, 100 μL of serum sample or standard was added to each well and incubated for 150 min at room temperature. Then, after four buffer washings (300 µL), 100 μL of detection anti-BNDF antibody was added to each well and incubated for 60 min at room temperature, followed by another four washes. Subsequently, 100 μL of HRP–avidin working solution were added to each well and incubated for 45 min at room temperature. After four washes, 100 μL of TMB One-Step Substrate Reagent were launched into each well and incubated for 30 min at room temperature. Then, 50 μL of stop solution were added to each well to terminate the color reaction. BDNF levels were measured using an MC Thermo Fisher Scientific reader (Thermo Fisher Scientific Inc., Helsinki, Finland) with an absorbance of 450 nm. The standard curve was used for the calculation of the relationship between the optical density and the BDNF. The BDNF contents are presented as pg/mg of tissue. The sensitivity and detection range of the BDNF rat ELISA kits were 0.051 ng/mL and 0.083–2.5 ng/mL. All samples were measured in duplicates.

### 4.8. Endocannabinoid Levels in Blood

Extraction and analysis of Endocannabinoids was performed as in [92], with some modifications: rat’s serum (100 μL each) was extracted with 1 mL of a pre-cooled (−20 °C) homogenous methanol: methyl-tert-butyl-ether (MTBE) 1:3 (*v/v*) mixture, containing following internal standards: 0.1 μg*mL^−1^ of Phosphatidylcholine (17:0/17:0) (Avanti), 0.4 μg*mL^−1^ of Phosphatidylethanolamine (17:0/17:0) (Avanti) and Methanandamide, 2 μg*mL^−1^. The tubes were vortexed and then sonicated for 30 min in an ice-cold sonication bath (taken for a brief vortex every 10 min). Then, UPLC-grade water: methanol (3:1, *v/v*) solution (0.5 mL) was added to the tubes followed by centrifugation. The upper organic phase was transferred, and the polar phase was re-extracted as described above, with 0.5 mL of MTBE. Both organic phases were combined and dried in speedvac and then stored at −80 °C. For analysis, the dried lipid extracts were re-suspended in 100 μL mobile phase B (see below) and centrifuged again at 13,000 rpm at 4 °C for 10 min.

Endocannabinoids were measured by UPLC-ESI-MS/MS equipped with the Acquity UPLC I class system (Waters). The MS detector (Waters Xevo TQ-XS) was equipped with an ESI source. The measurement was performed in positive ionization mode using MRM. MS parameters were as follows: the source and de-solvation temperatures were maintained at 150 °C and 400 °C, respectively. The capillary voltage was set to 1.5 kV. Nitrogen was used as de-solvation gas and cone gas at the flow rate of 800 L*h^−1^ and 150 L*h^−1^, respectively.

Chromatographic separation was performed on an ACQUITY UPLC BEH C8 column (2.1 × 100 mm, i.d., 1.7 μm) (Waters Corp., Milford, MA, USA). The mobile phase A consisted of 45% water (UPLC grade) with 1% 1 M NH4Ac, 0.1% acetic acid, and with 55% acetonitrile: isopropanol (7:3) with 1% 1 M NH4Ac, 0.1% acetic acid (mobile phase B). The column was maintained at 40 °C and the flow rate of the mobile phase was 0.4 mL*min^−1^. Mobile phase A was run for 1 min at 100%, then gradually reduced to 25% at 12 min, following a decrease to 0% at 16 min. Then, mobile phase B was run at 100% until 21 min, and mobile phase A was set to 100% at 21.5 min. Finally, the column was equilibrated at 100% until 25 min.

### 4.9. Western Blotting

#### Protein Extraction

Hippocampal punches (n = 4 or 5 per group, average weight = 4.81 mg ± 0.67) were placed in RIPA lysis buffer (50 mM tris, 0.5% Na-deoxycolate, 2% SDS, 1% NP-40/triton) diluted with protease inhibitor at 1:100, at a ratio of 5 mg/300 mL. Samples were then homogenated, then cooled on ice and centrifuged at 12,000 rpm for 20 min at 4 °C and protein lysates were transferred to fresh tubes. Protein concentration was determined by BCA protein assay (Thermo scientific).

### 4.10. Analysis of Proteins

Protein lysates were diluted in sample buffer (×4, 300 mM Tris-HCl pH = 6.8, 10% SDS, 50% glycerol, 0.03% bromophenol blue, 500 mM DTT), at a final volume of 20 μL with total amount of 10 μg protein for each well, loaded onto 10% Bis/Acrylamide gel, and standard electrophoresis was performed. Separated proteins were blotted onto Polyvinylidene fluoride (PVDF, Invitrogen, USA) membranes using a wet blotter apparatus for 70 min on 100 V on ice. The membrane was then blocked using 5% skim milk diluted in 0.1% (*v*/*v*) PBS-Tween20 (P9416-50 ML, Sigma, St. Louis, MO, USA) for 1 h at room temperature followed by overnight incubation at 4 °C with primary antibody diluted to 1:1000 in blocking buffer (mAb Mouse anti-FAAH1 [4H8] ab45615, (abCam). Blocking buffer and antibodies were prepared in 5% skim-milk in PBS-Tween. After incubation, membranes were washed 3 × 10 min in PBST, incubated with HRP-conjugated goat anti mouse IgG secondary antibody (Cat# 115-035-003, Peroxidase AffiniPure Goat Anti-Mouse IgG, Jackson immunoreasearch, PA, USA) diluted at 1:10,000 in blocking buffer for 1 h at room temperature), washed again and finally were developed with ECL kit (Bio-Rad Laboratories. Inc., CA, USA). The membrane was then exposed with a MicroChemi2 imager (DNR Bio-Imaging Systems, Israel). After exposure, protein levels were quantified by ImageJ 1.53e software (NIH, USA), and normalized by β-actin.

### 4.11. Statistical Analyses

The FST included two criteria; floating (immobility) and swimming. Therefore, one-way MANOVA was employed with a significance level of 0.05. The independent variable was treatment (dose/vehicle). To examine sex-differences, this MANOVA was repeated with the addition of sex as another between-subject factor. For the biological measures, MANOVA or ANOVA were employed where appropriate.

FAAH hippocampal levels were analyzed in two separated one-way ANOVAs as analysis of proteins with western blot is possible with up to 15 samples. The first ANOVA included samples from the treatment groups: CBDA-ME, AM-251 and vehicle, and the second included the treatment groups: CBDA-ME, AM-630 groups and vehicle.

Post-hoc Dunnett’s test was conducted to detect differences between the treatment groups. Statistical analyses were performed using SPSS 20.0 software (IBM Corp., Armonk, NY, USA).

One behavioral outlier (standard deviations > 2) was excluded from the analysis.

## 5. Conclusions

This study provides support for the potential benefits of the synthetic analogue of cannabidiolic acid, CBDA-ME, in treating depression, while highlighting the dimorphic response to its anti-depressive-like effect.

Furthermore, we suggest possible mechanisms that may be involved in this effect: mediation through CB2 receptors, in females only, by higher BDNF levels, as well as lower hippocampal FAAH expression and higher endocannabinoids’ blood levels.

Nevertheless, the cumulative data indicate that these pathways are still ambiguous and require future research in order to fully understand the mechanisms of action of acute CBDA-ME in relieving the symptoms of depression.

## Data Availability

The data that support the findings of this study are available from the corresponding author upon reasonable request.

## Supplementary Materials

The following supporting information can be downloaded at: https://www.mdpi.com/article/10.3390/ijms24043828/s1.
