# Supplementary material for: Cannabinoid Receptor 2 Blockade Prevents Anti-Depressive-like Effect of Cannabidiol Acid Methyl Ester in Female WKY Rats
Source: Int J Mol Sci. 2023 Feb 14;24(4):3828. doi: 10.3390/ijms24043828 (PMC9958868; doi:10.3390/ijms24043828)
Supplement: Supplementary file 1 [file ijms-24-03828-s001.zip › ijms-2095270-supplementary.pdf]

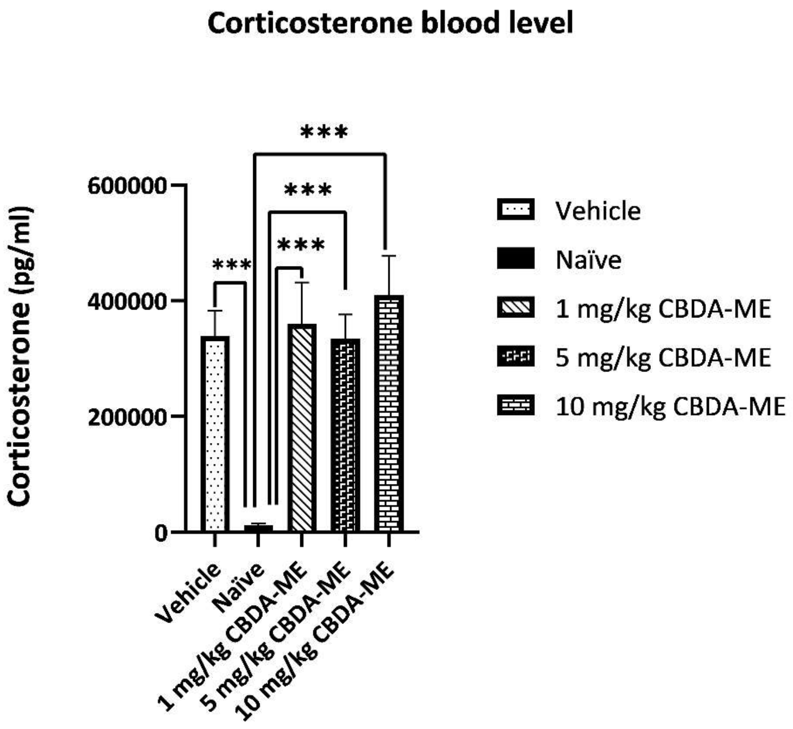

<u>Figure S1:</u> Corticosterone blood levels (mean + SEM) of female WKY rats. Rats ingested either vehicle (n = 12) or 1 mg/kg CBDA-ME (n = 12), 5 mg/kg CBDA-ME (n = 12), 10 mg/kg CBDA-ME (n = 12), Naïve (n = 12). *** p < .0.001.

<u>Table S1</u>: Cytokines blood levels (mean ± SD) of female WKY rats (n =8 in each group) that received different oral doses (0, 1, 5, 10 mg/kg) of CBDA-ME and naïve rats.

	**IFN gamma**	**IL-1 b**	**IL-2**	**IL-6**	**IL-10**	**TNF a**
**Naïve**	1914.875 (144.345)	46.6262 (3.8675)	100.24 (53.9101)	418.51875 (16.500)	89.35625 (7.89212)	557.6362 (25.3899)
**Vehicle**	1806.375 (185.350)	47.7475 (2.5938)	76.53625 (3.9036)	414.64125 (18.1051)	88.74125 (3.5538)	550.4475 (4.4923)
**1 mg/kg CBDA-ME**	1667.625 (203.899)	47.27 (1.5298)	75.755 (4.5744)	403.0025 (17.89042)	89.52875 (3.71367)	563.0562 (21.6646)
**5 mg/kg CBDA-ME**	1733.875 (241.1919)	46.95 (2.0238)	75.64625 (3.539)	406.4125 (13.76346)	88.06375 (6.1604)	551.0875 (10.7425
**10 mg/kg CBDA-ME**	1697.75 (230.906)	46.79 (0.82031)	75.98 (3.03109)	406.405 (16.09276)	89.40375 (2.3618)	552.455 (7.6266)

Table S2: FST data (mean ± SD) of male WKY rats (n = 12–13 in each group) in different treatment groups (0, 1 mg/kg CBDA-ME, 1 mg/kg AM251 + 1 mg/kg CBDA-ME, 1 mg/kg AM630 + 1 mg/kg CBDA-ME, 30 mg/kg imipramine).

	**Floating**	**Struggling**	**Swimming**
**Vehicle**	218.1818 (41.645)	12.8182 (13.702)	69.0000 (43.575)
**1 mg/kg CBDA-ME**	183.2727 (30.414)	12.9091 (10.123)	103.8182 (22.701)
**1mg/kg AM251+ 1 mg/kg CBDA-ME**	175.2500 (74.896)	16.2500 (14.181)	108.5000 (67.763)
**1mg/kg AM630+ 1 mg/kg CBDA-ME**	169.4167 (71.828)	14.1667 (16.0954)	72.1667 (65.7196)
**30 mg/kg imipramine**	231.5455 (50.301)	18.5000 (12.760)	112.0833 (63.950)

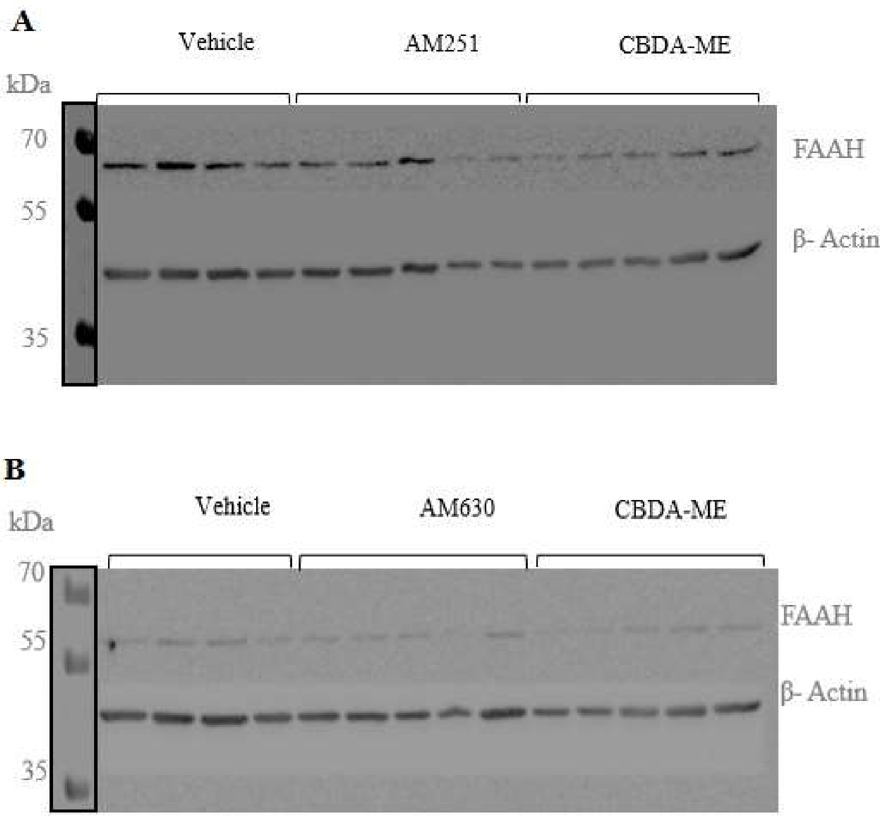

Figure S2: FAAH expression in the hippocampus of female WKY rats, images by western blot. Rats received either (A) vehicle (n = 4), 5mg/kg CBDA-ME (n = 5), or 1mg/kg AM251 + 5 mg/kg CBDA-ME (n = 5); (B) vehicle (n = 4), 5mg/kg CBDA-ME (n = 5), or 1mg/kg AM630 + 5 mg/kg CBDA-ME (n = 5).
